# COVID-19 vs. Classical Myocarditis Associated Myocardial Injury Evaluated by Cardiac Magnetic Resonance and Endomyocardial Biopsy

**DOI:** 10.3389/fcvm.2021.737257

**Published:** 2021-12-24

**Authors:** Radu Tanacli, Patrick Doeblin, Collin Götze, Victoria Zieschang, Alessandro Faragli, Christian Stehning, Grigorios Korosoglou, Jennifer Erley, Jakob Weiss, Alexander Berger, Felix Pröpper, Fridolin Steinbeis, Titus Kühne, Franziska Seidel, Dominik Geisel, Thula Cannon Walter-Rittel, Philipp Stawowy, Martin Witzenrath, Karin Klingel, Sophie Van Linthout, Burkert Pieske, Carsten Tschöpe, Sebastian Kelle

**Affiliations:** ^1^Department of Cardiology, German Heart Centre Berlin, Berlin, Germany; ^2^Department of Cardiology, Charité University Medicine Berlin, Charité-Universitätsmedizin Berlin, Berlin, Germany; ^3^Philips Healthcare Systems, Hamburg, Germany; ^4^Department of Cardiology, GRN Hospital Weinheim, Weinheim, Germany; ^5^German Centre for Cardiovascular Research DZHK, Partner Site Berlin, Berlin, Germany; ^6^Department of Infectious Diseases and Respiratory Medicine, Charité-Universitätsmedizin Berlin, Berlin, Germany; ^7^Institute for Imaging Science and Computational Modelling in Cardiovascular Medicine, Charité-Universitätsmedizin Berlin, Berlin, Germany; ^8^Department of Radiology, Charité-Universitätsmedizin Berlin, Berlin, Germany; ^9^Cardiopathology, Institute for Pathology and Neuropathology, University Hospital Tübingen, Tübingen, Germany; ^10^Berlin Institute of Health Center for Regenerative Therapies (BCRT), Charité-Universitätsmedizin Berlin, Berlin, Germany

**Keywords:** COVID-19, myocarditis, Lake Louise Criteria, CMR, biopsy, inflammation

## Abstract

**Background:** Despite the ongoing global pandemic, the impact of COVID-19 on cardiac structure and function is still not completely understood. Myocarditis is a rare but potentially serious complication of other viral infections with variable recovery, and is, in some cases, associated with long-term cardiac remodeling and functional impairment.

**Aim:** To assess myocardial injury in patients who recently recovered from an acute SARS-CoV-2 infection with advanced cardiac magnetic resonance imaging (CMR) and endomyocardial biopsy (EMB).

**Methods:** In total, 32 patients with persistent cardiac symptoms after a COVID-19 infection, 22 patients with acute classic myocarditis not related to COVID-19, and 16 healthy volunteers were included in this study and underwent a comprehensive baseline CMR scan. Of these, 10 patients post COVID-19 and 13 with non-COVID-19 myocarditis underwent a follow-up scan. In 10 of the post-COVID-19 and 15 of the non-COVID-19 patients with myocarditis endomyocardial biopsy (EMB) with histological, immunohistological, and molecular analysis was performed.

**Results:** In total, 10 (31%) patients with COVID-19 showed evidence of myocardial injury, eight (25%) presented with myocardial oedema, eight (25%) exhibited global or regional systolic left ventricular (LV) dysfunction, and nine (28%) exhibited impaired right ventricular (RV) function. However, only three (9%) of COVID-19 patients fulfilled updated CMR–Lake Louise criteria (LLC) for acute myocarditis. Regarding EMB, none of the COVID-19 patients but 87% of the non-COVID-19 patients with myocarditis presented histological findings in keeping with acute or chronic inflammation. COVID-19 patients with severe disease on the WHO scale presented with reduced biventricular longitudinal function, increased RV mass, and longer native T1 times compared with those with only mild or moderate disease.

**Conclusions:** In our cohort, CMR and EMB findings revealed that SARS-CoV-2 infection was associated with relatively mild but variable cardiac involvement. More symptomatic COVID-19 patients and those with higher clinical care demands were more likely to exhibit chronic inflammation and impaired cardiac function compared to patients with milder forms of the disease.

## Background

The systemic (immune) response to a SARS-CoV-2 infection varies widely, ranging from asymptomatic or mildly symptomatic respiratory infection to a systemic life-threatening condition with multiple organ failure. A three-phase model of pathogenesis of COVID-19 has been proposed (1) where a significant minority of patients progress to a critical hyperinflammation phase characterized by a systemic host response with elevated IL-2, IL-6, IL-7, TNF-α, C-reactive protein, and D-dimer levels. Several studies (2–4) demonstrated cardiac pathologic modifications reflected by elevated troponin and N-terminal pro B-type natriuretic peptide in 10–28% of COVID-19 patients, requiring hospitalization. In a large multicentre study (5), myocardial injury was diagnosed in 62% of cases presenting with troponin elevation and was associated with higher percentage of abnormal echocardiographic findings and higher mortality. Moreover, patients with cardiovascular comorbidities are more likely to develop severe forms of COVID-19 (2, 3).

Several pathogenic mechanisms may explain the specific cardiac findings post-COVID-19: triggered pan-endotheliitis (4) or macrophage activation (6) precipitating acute plaque rupture and coronary events (7), imbalanced activation of helper T cells leading to cytokine storm and direct myocardial injury (3), sepsis, or hypoxia-induced myocyte apoptosis (8).

A recent cardiac magnetic resonance (CMR) cross-sectional study (9) suggested that COVID-19 might be responsible for a sustained subacute or chronic inflammatory state of the myocardium comparable with cases of viral myocarditis and prone to cause long-term cardiac impairment by downstream activation of ventricular remodeling and fibrosis. However, the clinical relevance of these findings has been discussed controversially, given the lack of a matched comparison group (10). To date, studies comparing CMR findings including late gadolinium enhancement (LGE) and mapping with histology are limited, and longitudinal analyses are completely missing in this context (11).

In this study, we used an advanced CMR protocol to examine potential effects of COVID-19 on cardiac function and structural remodeling in consecutive patients with a recent SARS-CoV-2-infection using endomyocardial biopsy (EMB) data as the reference standard. In addition, we sought to compare these findings to healthy volunteers and a cohort of patients with “classic” myocarditis. Follow-up CMR assessment was performed in patients with COVID-19 and in those with “classic” myocarditis. To compare histological and/or immunohistological findings between patients with COVID-19 and patients with myocarditis, available EMB samples were evaluated according to the current diagnostic criteria for myocarditis (12).

## Methods

### Study Population

All post-COVID-19 patients referred to our clinic with a clinical indication for CMR (13) were asked for consent to be included in our observational study. Healthy control subjects were identified from an existing database available at our institution (13). This study was reviewed and approved by the Charité–Universitätsmedizin Berlin Ethics Committee and complies with the Declaration of Helsinki.

In total, 32 patients with a previous COVID-19 infection were included in the study. For comparison, we retrospectively included 22 patients with a clinically confirmed diagnosis of acute non-COVID-19 myocarditis and an available baseline CMR scan, along with 16 healthy volunteers. Ten of the post-COVID-19 patients underwent a clinically indicated follow-up scan. Thirteen of the 22 patients with acute myocarditis had an available follow-up CMR on file. Inclusion criteria for the post-COVID-19 patients were as follows: (I) a previously diagnosed (14) SARS-CoV-2 infection with COVID-19 disease; (II) clinical indication for CMR such as suspected myocardial injury (elevated troponin), reduced LVEF and/or presence of pericardial effusion on echocardiogram, persistent arrhythmia, persistent dyspnoea, reduced exercise capacity, or fatigue; and (III) resolution of acute COVID-related symptoms to allow the end of self-isolation or a confirmation of a negative PCR test. Exclusion criteria were absolute contraindications to CMR and impossibility to obtain consent. The non-COVID-19 myocarditis cases were retrospectively identified from our local database and inclusion criteria followed the most recent ESC recommendations (15). Exclusion criteria were as follows: coexistence of underlying cardiac pathology (myocardial infarction, cardiomyopathy, and/or haemodynamically relevant valvulopathy) (16).

### WHO Score Description

World Health Organization (WHO) guidance on clinical management of COVID-19 (https://www.who.int/publications/i/item/WHO-2019-nCoV-clinical-2021-2) was used to define disease severity as follows: mild and moderate disease–symptomatic patients meeting the case definition for COVID-19 without evidence of viral pneumonia, hypoxia, or some clinical signs of pneumonia (fever, cough, dyspnoea, and/or fast breathing) without other criteria of severity and SpO_2_ ≥ 90% on room air. Severe and critical disease were defined as presence of additional severity signs and respiratory distress as follows: respiratory rate > 30 breaths/min; severe respiratory distress; or SpO2 <90% at room air or presence of acute respiratory distress syndrome or specific signs on radiograph, CT scan, or lung ultrasound (e.g., bilateral opacities, not fully explained by volume overload, lobar or lung collapse, or nodules).

### Cardiac Magnetic Resonance

**Figure 1:**
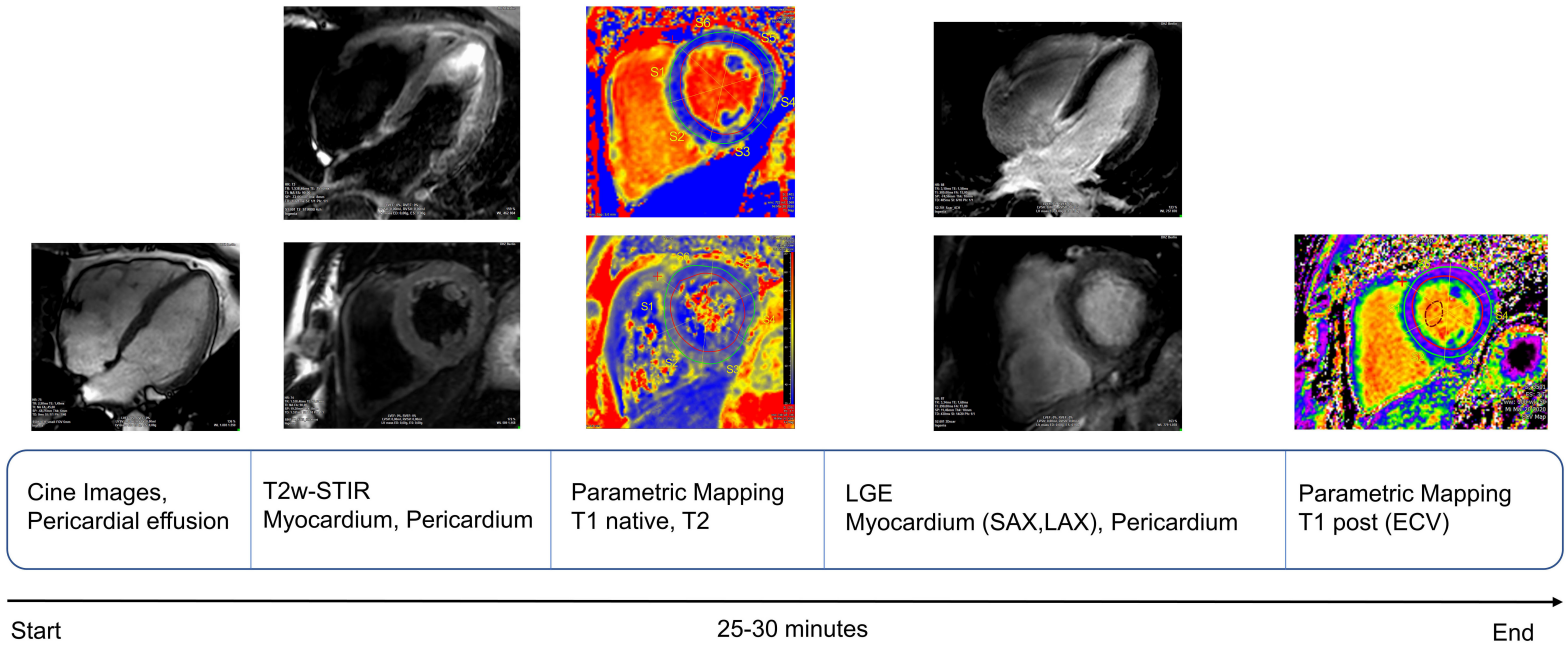
Schematic representation of cardiac magnetic resonance (CMR) protocol workflow used for our study, in keeping with recent SCMR recommendations for evaluation of patients with COVID-19 (18).

Cardiac magnetic resonance (CMR) images of the following patients were acquired using three clinical MRI scanners: 25 post-COVID-19 patients, 18 patients with myocarditis, and all 16 healthy controls with a clinical 3T system (Ingenia, Philips Healthcare, Best, the Netherlands), four post-COVID-19 patients, four patients with myocarditis with a 1.5T system (Achieva, Philips Healthcare, Best, the Netherlands), and three post-COVID-19 patients with a 1.5T system (Magnetom Aera, Siemens Inc.). All study participants were scanned with a comprehensive imaging protocol and appropriate local receiver coil arrays in accordance with the Society for Cardiovascular Magnetic Resonance (SCMR) guidelines (17) (Figure 1).

All CMR scans were acquired with ECG gating or retrospective gating (cine) in one breath hold (8–15 s). Typical imaging parameters are summarized as follows:

*Long-axis cine-imaging (Philips 3T):* balanced steady state free precession *(*bSSFP), TR = 2.9 ms, TE = 1.45 ms, flip angle = 45°, acquisition voxel size = 1.9 × 1.9 × 6.0 mm^3^, acquired/reconstructed heart phases = 27/40, parallel imaging (SENSE) acceleration = 2.

*Long axis cine-imaging (Philips 1.5T):* TR = 3.4 ms, TE = 1.7 ms, flip angle = 60°, acquisition voxel size = 1.6 × 1.6 × 6.0 mm^3^, acquired/reconstructed heart phases = 25/50, parallel imaging (SENSE) acceleration = 2.

*T2-weighted short tau inversion recovery STIR (Philips 1.5T / 3T):* black blood prepared turbo spin echo (TSE) imaging, TE = 100 ms, flip angle 90°, refocusing angle 160°, acquisition voxel size = 1.5 × 1.5 × 8.0 mm^3^, half-Fourier factor 0.65, acquisition every other heart beat to allow for optimal blood signal suppression.

*T2 mapping*: (Philips 1.5T / 3T): black blood prepared gradient and spin echo (GraSE) imaging, flip angle 90°, refocusing angle 180°, 9 echoes, echo times = n × 8.8 ms, EPI-factor 7, acquisition voxel size = 2.0 × 2.0 × 10.0 mm^3^, parallel imaging (SENSE) acceleration = 2.3.

*Siemens 1.5T Magnetom Aera*: repetition time (TR) = 40.8 ms, echo time (TE) = 1.07 ms, flip angle = 55°, acquisition voxel size = 1.9 × 1.9 × 8.0 mm^3^ (19).

Native and 15 min post-contrast T1-mapping were performed using modified Look-Locker imaging (MOLLI) in two left-ventricular short-axis slices (basal and mid-ventricle), as described previously (17). Patients received.15 mmol/kg of gadolinium-based contrast agent (Gadobutrol 1.0 mmol/ml, Gadovist®, Bayer AG, Leverkusen, Germany). Segmented inversion-recovery fast gradient–echo imaging was used to assess late gadolinium enhancement (LGE) 10 min after the administration of contrast substance (19). mDixon-imaging was used to differentiate pericardial enhancement from fat (20).

### Image Analysis

All images were analyzed offline by two cardiologists with more than 10 years of experience in CMR and are certified SCMR Level 3. We used commercially available software (Medis Suite, version 3.1, Leiden, The Netherlands) in accordance to a recent consensus paper for the quantification of left ventricular (LV) function in CMR (16) and our internal standard operating procedures (MRI Core Lab, German Heart Center, Berlin, Germany). To assess whether the updated Lake Louise Criteria (LLC) for myocarditis were fulfilled, the proposed updated analysis algorithm (21) was scrupulously followed.

Global myocardial longitudinal (GLS) and circumferential (GCS) strain was assessed at the level of 2 distinct myocardial layers: Endo (subendocardial layer) and Myo (midwall layer) as previously described (17). Similarly, right ventricular (RV) GLS at Endo and Myocardium levels was determined through drawing RV endocardial and epicardial contours in 4Ch cine images with automatic propagation over the whole cardiac cycle using QStrain (22). Similarly, left atrial (LA) strain was measured in 4Ch and 2Ch views and these values averaged. Mapping parameters were measured using QMap RE version 2.0 (Medis Medical Imaging Systems bv, Leiden, the Netherlands). For parametric imaging, pre- and post-contrast MOLLI images were manually adjusted for in-plane motion and T1 native and post-contrast relaxation times were determined using nonlinear fitting with a maximum likelihood estimator (17). Extracellular volume (ECV) was computed from pre- and post-contrast T1 and hematocrit values as previously described (23). Given the inherent variability of normal values in parametric imaging between different field strengths and magnets (24), we only present the parametric values acquired on the 3T Ingenia scanner. Healthy controls received no contrast and for comparison, ECV reference values (24) corresponding to the same model of scanner and magnetic field strength were used. Global T1 native, ECV, and T2 values were calculated by averaging individual segmental values derived for each patient from mapping of two distinct ventricular short-axis (SA) slices at basal and mid-ventricular levels (17). The presence of LGE was established by visual assessment of two experienced readers (consensus read, both CMR-level-III certified), and evaluated in all the slices of the short axis stack and three long axis views.

### Biopsy Samples

In all patients, including those with COVID-19 undergoing EMB, myocarditis was clinically suspected, following the recent guideline recommendations (15). Myocardial biopsy was performed in all COVID-19 and myocarditis cases as described previously. Briefly, endomyocardial samples were collected through femoral vein access using either a 7F long-sheath with angulated tips (from the RV surface of the interventricular septum) or a 7F long-sheath without angulation using a retrograde approach (from the LV surface of the interventricular septum). At least four pieces per patient were collected with which Fluoroscopic guidance was used to identify the region of interest. Vital parameters and ECG were closely monitored during the procedure. A routine echocardiogram was performed at the end of the procedure to exclude iatrogenic pericardial effusion. Analysis of endomyocardial biopsy (EMB) samples was performed in specialized laboratories by experienced cardio-pathologists as described previously (25). Myocardial inflammation was considered to be present when ≥ 20 infiltrating immune cells/mm^2^ were observed (CD3 T-lymphocytes and/or CD68 macrophages). Additionally, enhanced HLA class II expression in antigen-presenting immune cells was evaluated. Screening for viral genomes was performed after extraction of deoxyribonucleic acid (DNA) and ribonucleic acid (RNA) with Proteinase-K digestion and phenol/chloroform. Reverse transcriptase-polymerase chain reaction was used subsequently to detect virus presence within cardiac tissue samples, e.g., COVID-19 and, respectively, viruses frequently involved in myocarditis, such as enteroviruses (including coxsackieviruses of group A and B and echoviruses), parvovirus B19 (PVB19), adenoviruses, human cytomegalovirus, Epstein-Barr virus, and human herpesvirus type 6 (HHV6). Oligonucleotide sequences were chosen from the glyceraldehyde-3-phosphate-dehydrogenase gene as a control for successful extraction of DNA and RNA. Negative and positive controls were included in each PCR reaction. Automatic DNA sequencing was used to confirm the specificity of all viral amplification products. Masson trichrome staining was used for histological examination of various types of fibrosis (multifocal fibrosis/scarring without inflammation/diffuse collagen deposition). Congo-red staining was used to exclude amyloid deposition.

### Statistical Analysis

All the data within the text, tables, or figures is presented as mean ± SD unless stated otherwise. The Shapiro-Wilk test was used to assess normal distribution of data for every dataset included. To compare the three subgroups, a one-way ANOVA for normally distributed data or Kruskal-Wallis for non-normally distributed data was performed followed by Tukey *post-hoc* tests to compare differences among subgroups. The Wilcoxon test was used to assess the differences between baseline and follow-up groups. The Welch correction was applied for unequal sample sizes or if the assumption of homogeneity of variance was infirmed. For differences between categorical variables, Fisher's exact and χ^2^ test were used. A two-tailed *p*-value below.05 was considered statistically significant. All statistical tests were performed using SPSS version 27.0.

## Results

### Study Population Characteristics

Demographics, clinical, and biochemical characteristics of the patients included in our study are presented in Table 1. Twelve (38%) of 32 patients with COVID-19 and 17 (77%) of 22 patients with non-COVID-19 “classic” myocarditis required hospitalization. According to the WHO disease severity criteria for COVID-19, 20 out of 32 (63%) patients with COVID-19 had mild or moderate, seven (22%) had severe, and five (16%) had critical disease. While fever, cough, and loss of taste or smell were dominant in COVID-19 patients, some of them also suffered from palpitations (1, 3%), chest pain *(*8, 25%), arrhythmia (9, 28%), or had elevated troponin (9, 28%) or NT-proBNP (6, 24%). During the convalescence phase, fatigue persisted in 9 (28%), loss of taste or smell in 2 (6%), and amnesia in two (6%) patients with COVID-19.

**Table 1:** Demographics clinical parameters.

	**Healthy Control** ***N*** **= 16**	**COVID-19** ***N*** **= 32**	**Myocarditis** ***N*** **= 22**	**P ANOVA**	**P**	**P**	**P**
					**Control vs. COVID-19**	**Control vs. Myocarditis**	**COVID-19 vs. Myocarditis**
**Patient characteristics**							
Age, years	24 ± 5	48 ± 14	32 ± 15	*** <0.001***	*** <0.001***	***0.031***	***0.004***
Male, *N* (%)	8 (50)	19 (59)	17 (77)		0.54	0.08	0.17
BMI	22 ± 3	26 ± 5	26 ± 5	***0.005***	***0.004***	***0.030***	0.75
Hypertension, *N* (%)	0 (0)	13 (42)	3 (19), *N =* 16		***0.012***	0.12	0.14
Diabetes, *N* (%)	0 (0)	1 (3)	1 (6), *N =* 16		0.48	0.31	0.22
Hypercholesterolemia, *N* (%)	0 (0)	8 (26)	2 (13), *N =* 16		***0.029***	0.14	0.31
Known CAD, *N* (%)	0 (0)	3 (10)	1 (6), *N =* 16		0.21	0.31	0.71
Smoking, *N* (%)	0 (0)	13 (41)	5 (31), *N =* 16		***0.003***	***0.015***	0.53
COPD or asthma, *N* (%)	0 (0)	3 (10)	2 (13), *N =* 16		0.21	0.14	0.74
Systolic Blood pressure, mm Hg	112 ± 17	119 ± 15	114 ± 19		0.50	0.92	0.72
Diastolic Blood pressure, mm Hg	68 ± 11	73 ± 11	66 ± 10		0.26	0.90	0.09
Heart rate, beats per min	65 ± 5	78 ± 15	88 ± 22		0.83	0.94	0.91
**Blood test results**							
High-sensitivity CRP, mg/dL		4 ± 9, *N =* 23	8 ± 12, *N =* 18				0.17
Elevated hsCRP, *N* (%)		5 (22), *N =* 23	10 (56), *N =* 18				***0.033***
Elevated Troponin, *N* (%)		9 (45), *N =* 20	10 (71), *N =* 14				0.17
CK, U/L		70 ± 37, *N =* 22	343 ± 396, *N =* 20				***<0.001***
CK-MB, U/L		18 ± 5, *N =* 15	37 ± 25, *N =* 20				***<0.001***
NT-proBNP, pg/mL		1291 ± 2484, *N =* 23	2194 ± 2360, *N =* 17				0.19
Elevated NT-proBNP, *N* (%)		6 (24), *N =* 25	15 (88), *N =* 17				***<0.001***
eGFR, mL/min		83 ± 24, *N =* 26	95 ± 16, *N =* 17				***0.045***
**Medication**		***N** **=*** **32**	***N** **=*** **16**				
Oral Anticoagulants, *N* (%)		4 (13)	2 (13)				0.70
Statins, *N* (%)		3 (9)	1 (6)				0.51
β-blockers, *N* (%)		14 (44)	8 (50)				0.59
Diuretics, *N* (%)		12 (38)	6 (38)				0.43
Nitrates, *N* (%)		0 (0)	0 (0)				0.99
ACE inhibitors, *N* (%)		10 (31)	3 (19)				0.14
Sartans, *N* (%)		5 (16)	1 (6)				0.20
Calcium Antagonists, *N* (%)		2 (6)	0 (0)				0.23
**Symptoms**		***N** **=*** **32**	***N** **=*** **16**				
Initial Presentation							
Fever, *N* (%)		19 (59)	5 (31)				0.07
Chest pain, *N* (%)		8 (25)	9 (56)				***0.033***
Dyspnea, *N* (%)		20 (63)	9 (56)				0.68
Arrythmia, *N* (%)		1 (3)	7 (44)				***<0.001***
Cough, *N* (%)		24 (75)	1 (6)				***<0.001***
Nausea/vomiting/diarrhea, *N* (%)		11 (34)	3 (19)				0.26
Fatigue/weakness, *N* (%)		24 (75)	11 (69)				0.65
Amnesia, *N* (%)		10 (31)	5 (31)				0.99
Lack of taste or smell, *N* (%)		21 (66)	0 (0)				***<0.001***
Persistent							
Fatigue/weakness, *N* (%)		9 (28)	0 (0)				***0.019***
Amnesia, *N* (%)		2 (6)	0 (0)				0.31
Lack of taste or smell, *N* (%)		2 (6)	0 (0)				0.31
Arrythmia, *N* (%)		9 (28)	3 (19)				0.48

*BMI, body mass index; CAD, coronary artery disease; COPD, chronic obstructive pulmonary disease; COVID-19 coronavirus disease; hsCRP, high sensitivity C-reactive protein; CK, creatin-kinase; NT- proBNP, N-terminal pro-b type natriuretic peptide; eGFR, estimated glomerular filtration rate; ACE, acetyl coenzyme A*.

*For comparison of the continuous variables, ANOVA and post-hoc Tukey's tests were used, for categorial variables Chi-square or Fischer tests test were used, a P < 0.05 was considered significant. For incomplete set of data, N represents the number of subjects included in the analysis. Statistically significant p-values are indicated in bold*.

Post-COVID-19 patients were scanned for a baseline visit at 95 ± 59 days after a first positive test and received a follow-up scan 68 ± 40 days after the baseline scan. Patients with myocarditis were scanned shortly after the onset of symptoms had a longer and more variable follow-up interval of 156 ± 124 days after baseline. There was an age discrepancy between the healthy control, post-COVID-19. and myocarditis groups (24 ± 5 vs. 48 ± 14 vs. 32 ± 15 years, *p* < 0.001). Healthy controls had a lower BMI than post-COVID-19 and patients with myocarditis. The risk factors were similar in the post-COVID-19 and myocarditis groups (Table 1).

### CMR Parameters: Comparison Between COVID-19, Myocarditis, and Healthy Volunteers

Cardiac magnetic resonance (CMR) parameters for each of the three groups are presented in Table 2. In one of the COVID-19 patients, no contrast agent was administered due to claustrophobia and distress during the scan. Hence, an abbreviated protocol was used in this case. Given the discrepancies between the normal range of values for parametric mapping (T1, ECV, T2 values) between different field strengths and scanners, only the patients and volunteers scanned in the 3T Philips Ingenia scanner were included in the analysis (specific numbers per group are specified in brackets in Table 2).

**Table 2:** Cardiac magnetic resonance imaging findings. baseline.

	**Healthy Control**	**COVID−19**	**Myocarditis**	**P ANOVA**	**P**	**P**	**P**
					**Control vs. COVID-19**	**Control vs. Myocarditis**	**COVID-19 vs. Myocarditis**
	***N =*** **16**	***N =*** **32**	***N =*** **22**				
**left Ventricle**							
ED volume, mL/m2	82 ± 9	78 ± 25	104 ± 31	***0.001***	0.94	***0.022***	***<0.001***
ES volume, mL/m2	33 ± 4	32 ± 20	54 ± 34	***0.003***	0.98	***0.023***	***0.004***
Stroke volume, mL/m2	49 ± 7	48 ± 8	50 ± 11	0.66			
Ejection fraction, %	60 ± 4	62 ± 10	52 ± 16	***0.006***	0.77	0.09	***0.005***
Cardiac Index, L/min/m2	3.5 ± 0.8	3.6 ± 0.9	3.7 ± 0.9	0.80			
Endo Longitudinal Strain %	−26.0 ± 2.1	−24.2 ± 4.4	−20.1 ± 7.0	***0.001***	0.61	***0.003***	***0.008***
Myo Longitudinal Strain %	−24.5 ± 2.0	−22.3 ± 4.1	−18.3 ± 5.8	***<0.001***	0.25	***<0.001***	***0.005***
Endo Circumferential Strain %	−31.5 ± 4.4	−32.0 ± 7.2	−24.0 ± 9.3	***<0.001***	0.96	***0.009***	***<0.001***
Myo Circumferential Strain %	−20.9 ± 2.7	−20.9 ± 4.2	−17.3 ± 6.9	***0.025***	0.99	0.08	***0.030***
LV Mass (g/m2)	50 ± 7	55 ± 19	72 ± 23	***<0.001***	0.70	***0.001***	***0.003***
**left Atrium**							
LA max Vol mL	37 ± 9	36 ± 8	41 ± 11	0.26			
LA emptying fraction %	70 ± 6	61 ± 12	54 ± 17	***0.003***	0.07	***0.002***	0.19
LA strain %	44 ± 11	39 ± 12	28 ± 16	***0.001***	0.42	***0.001***	***0.011***
**Right Ventricle**							
ED volume, mL/m2	87 ± 10	77 ± 15	86 ± 26	0.10			
ES volume, mL/m2	38 ± 7	36 ± 10	42 ± 19	0.26			
Stroke volume, mL/m2	49 ± 8	41 ± 9	44 ± 12	***0.040***	***0.031***	0.30	0.51
Ejection fraction, %	56 ± 6	54 ± 8	53 ± 11	0.55			
Endo RV longitudinal strain %	−29.7 ± 7.1	−28.2 ± 7.8	−27.1 ± 7.3	0.56			
Myo RV longitudinal strain %	−27.9 ± 6.5	−26.0 ± 7.4	−25.2 ± 6.8	0.81			
RV Mass (g/m2)	13 ± 1	13 ± 4	16 ± 3	***0.006***	0.87	***0.013***	***0.014***
**Right Atrium**							
RA max Vol mL	40 ± 7	37 ± 12	43 ± 11	0.15			
RA emptying fraction %	56 ± 9	54 ± 11	47 ± 13	***0.027***	0.80	***0.039***	0.07
RA strain %	50 ± 16	42 ± 13	36 ± 13	***0.012***	0.16	***0.009***	0.25
**Parametric Imaging**							
T1 native	1236 ± 21 (*N =* 16)	1271 ± 50 (*N =* 25)	1352 ± 113 (*N =* 12)	***<0.001***	0.21	***<0.001***	***0.003***
ECV	26 ± 4^*^ (ref)	25 ± 4 (*N =* 24)	30 ± 8 (*N =* 12)	***0.044***	0.59	0.06	***0.035***
T2	43 ± 2 (*N =* 16)	48 ± 6 (*N =* 25)	61 ± 10 (*N =* 15)	***<0.001***	***0.026***	***<0.001***	***<0.001***
		***N** **=*** **31**	***N** **=*** **22**				
**LGE No. (%)**							
Ischaemic	n/a	1 (3)	3 (14)				0.17
Nonischaemic	n/a	5 (16)	19 (86)				***<0.001***
Pericardial	n/a	3 (10)	4 (18)				0.37
^*^ Reference Value							

*ED, end diastolic; ES, end systolic; Endo, subendocardial layer; Myo, mid-myocardial layer; LV, center ventricular, RV, right ventricular; LA, center atrial; RA, right atrial; ECV, extracellular volume; LGE, late gadolinium enhancement*.

*For comparison of the continuous variables, ANOVA and post-hoc Tukey's tests were used, for categorial variables Chi-square or Fischer tests test were used, a P <0.05 was considered significant. For incomplete set of data, N represents the number of subjects included in the analysis*.

* *Reference Values for ECV in healthy controls was taken from Dabir et al., (24). Statistically significant p-values are indicated in bold*.

**Figure 2:**
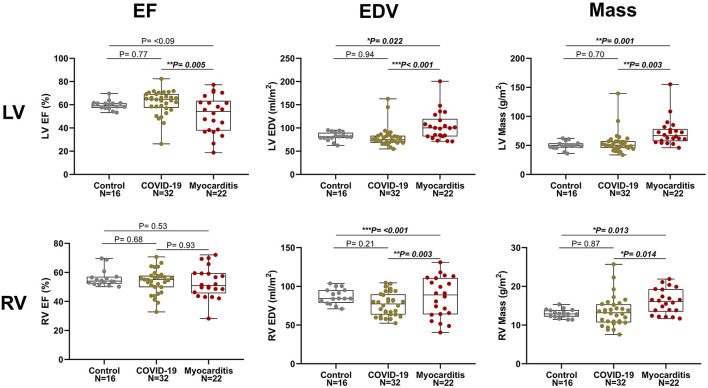
Left ventricle (LV, upper row) and right ventricular (RV, lower row) ejection fraction *(*EF), end diastolic volume (EDV), and myocardial mass in three pathology groups (from left to right: Control, COVID-19, and Myocarditis). There is no difference on average between patients with COVID-10 and Controls. However, there is significant dysfunction and remodeling in some of these patients that overlaps with the myocarditis spectrum. A *p* < 0.05 was considered statistically significant, and indicated as follows: ^*^ < 0.05, ^**^ < 0.01. ^***^ < 0.001.

There were no significant differences between post-COVID-19 and controls for any of the CMR parameters apart from T2 values, which were significantly higher in the COVID-19 (48 ± 6 vs. 43 ± 2 ms, *p* = 0.026). Compared to controls, RV stroke volume was significantly lower in the COVID-19 group (41 ± 9 vs. 49 ± 8 ml/m^2^, *p* = 0.031). There were no differences between LV function, RV function, and mass between COVID-19 patients and healthy controls (Figure 2).

**Figure 3:**
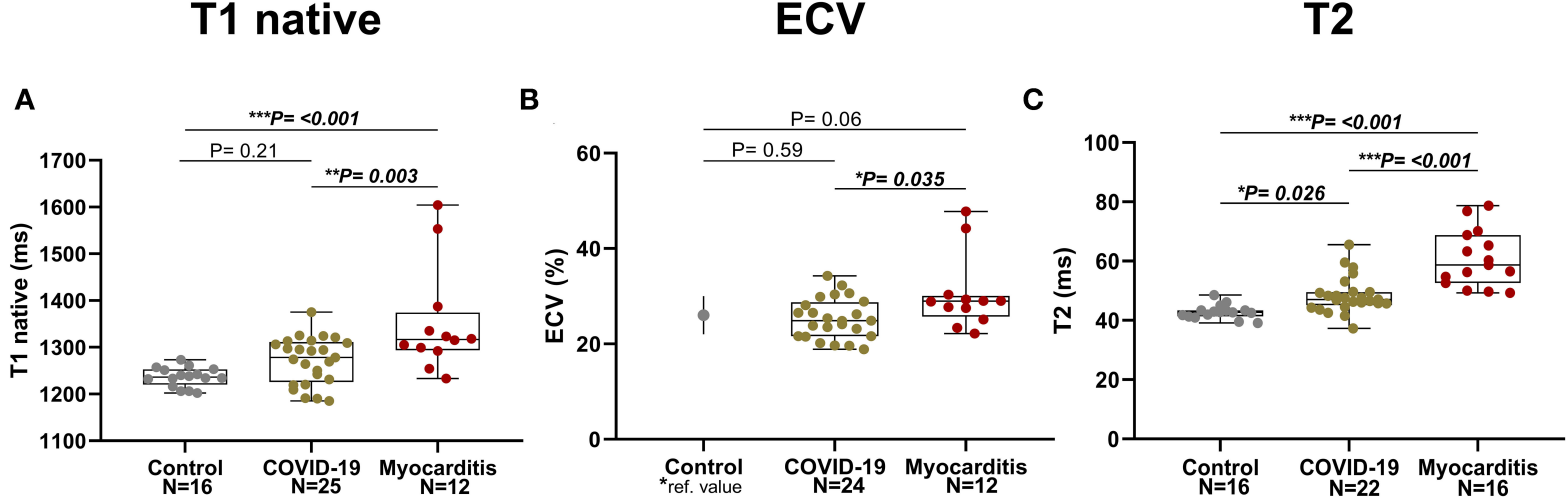
Parametric Imaging in three pathology groups (from left to right: Control, COVID-19, Myocarditis): **(A)** T1 native, **(B)** Extracellular volume (ECV)–for the Control it is represented the mean and SD referenced in literature [Dabir et al., (24)] corresponding to similar manufacturer and magnet field strength, **(C)** T2. A *p* < 0.05 was considered statistically significant, and indicated as follows: ^*^ < 0.05, ^**^ < 0.01, ^***^ < 0.001.

Patients with COVID-19 had a normal left ventricular ejection fraction *(*LVEF), compared to patients with myocarditis who had significantly reduced LVEF (61 ± 11 vs. 51 ± 17%, *p* = 0.016). LV dysfunction in myocarditis was also reflected by lower values of longitudinal and circumferential strain, which were normal in patients with COVID-19. LV and RV mass were both increased in myocarditis but not in COVID-19 compared with controls (Figure 3). Atrial function, measured as LA and RA emptying fractions and strain, was impaired in myocarditis compared with controls and COVID-19. T1 native, ECV and T2 values were all higher in myocarditis compared with controls and COVID-19 (see Figure 3, Table 2). Myocardial and pericardial LGE was present in all patients with myocarditis, while only 6 (19%) of the patients with COVID-19 had myocardial and 6 (19%) had pericardial LGE (Table 2).

### CMR–Lake Louise Criteria and EMB Histological Analyses

**Figure 4:**
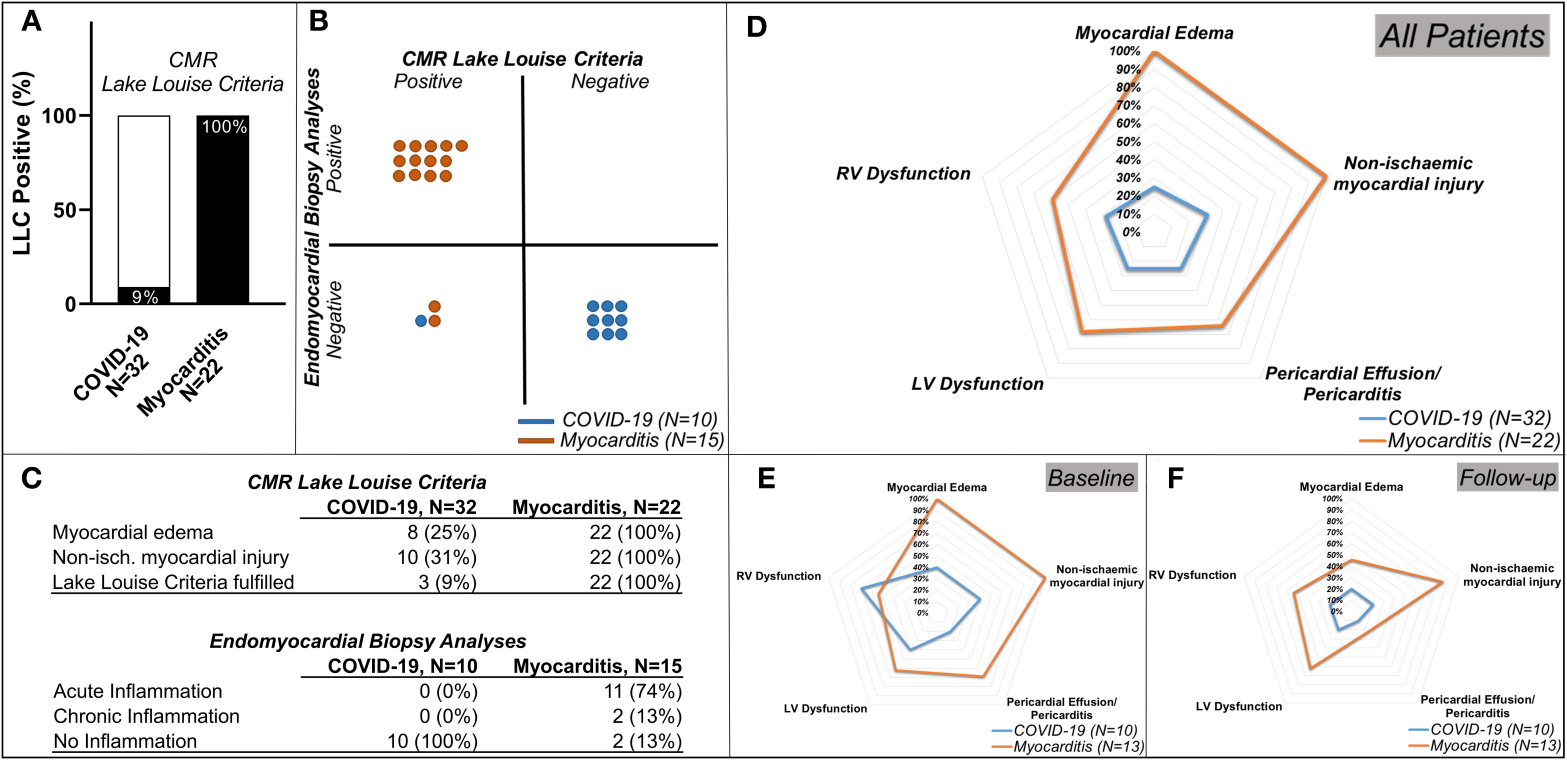
Lake Louise Criteria (LLC) and Functional Impairment in patients with COVID-19 and patients with Myocarditis. **(A)** Comparison of the CMR LLC and endomyocardial biopsy (EMB) findings. Three out of 32 (9%) patients with COVID-19 and all 22 (100%) of the patients with myocarditis have positive LLC, **(B)** from the patients in which endomyocardial biopsy (EMB) was available: none of the patients with COVID-19 and 13 (87%) out of 15 patients with myocarditis with available EMB had histological findings in keeping with this diagnosis, 1 (10%) of the patients with COVID-19, and 2 (13%) of the patients with myocarditis have positive LLC but negative EMC diagnostic criteria. **(C)** tables with numeric values for CMR LLC (upper rows) and Endomyocardial Biopsy Analyses (lower rows) **(D)** spidernet representation of myocardial inflammation, injury, pericardial involvement, LV, and RV dysfunctions in all COVID-19 (blue polygons) and all Myocarditis (red polygons) patients, **(E)** (baseline), **(F)** (follow-up)**–**spidernet representations of myocardial inflammation, injury, pericardial involvement, LV, and RV dysfunctions in patients with COVID-19 (blue polygons) and Myocarditis (red polygons) with an available follow-up scan.

In total, only three (9%) post-COVID-19 patients fulfilled the updated LLC for acute myocarditis, four (13%) presented signs of myocardia oedema (areas of elevated signal on T2 maps or T2-weighted fat suppressed images), and 10 (31%) had signs of myocardial injury (abnormal T1, ECV or LGE). Additionally, eight (25%) had LV wall motion abnormalities, nine (28%) had an impaired RV function, and eight (25%) had evidence of pericardial effusion and/or pericarditis (Figures 4A,C, Table 3).

**Table 3:** Summary of Lake Louise Criteria and Ventricular dysfunction.

**All patients, baseline**				
**A**	**COVID-19 (*****N*** **=** **32)**		**Myocarditis (*****N*** **=** **22)**	
2018 Lake Louise for myocarditis fulfilled N (%)	3 (9)		22 (100)	
Myocardial edema (T2-mapping or T2W images)	8 (25)		22 (100)	
Non-ischaemic myocardial injury (abnormal T1, ECV or LGE)	10 (31)		22 (100)	
Pericarditis (effusion in cine images or abnormal LGE, T2 STIR)	8 (25)		14 (64)	
Systolic LV dysfunction (regional and or global WMA)	8 (25)		15 (68)	
Depressed LVEF, *N* (%)	6 (19)		11 (50)	
LV dilatation, *N* (%)	3 (9)		13 (59)	
LV increased wall thickness, *N* (%)	7 (22)		4 (18)	
Depressed RVEF, *N* (%)	9 (28)		13 (59)	
RV dilatation, *N* (%)	4 (13)		9 (41)	
**Only patients will follow-up**				
**B**	**COVID-19 (*****N*** **=** **10)**		**Myocarditis (*****N*** **=** **13)**	
	**Baseline**	**Follow-up**	**Baseline**	**Follow-up**
2018 Lake Louise for myocarditis fulfilled, *N* (%)	2(20)	1(10)	13 (100)	5 (38)
Myocardial edema (T2-mapping or T2W images)	4 (40)	2 (20)	13 (100)	6 (46)
Non-ischaemic myocardial injury (abnormal T1, ECV or LGE)	4 (40)	2 (20)	13 (100)	11(85)
Pericarditis (effusion in cine images or abnormal LGE, T2 STIR)	2 (20)	1 (10)	9 (69)	3 (23)
Systolic LV dysfunction (regional and or global WMA)	4 (40)	2 (20)	8 (62)	8 (62)
Depressed LVEF, *N* (%)	3 (30)	1 (10)	5 (38)	4 (31)
LV dilatation, *N* (%)	1 (10)	0 (0)	7 (54)	4 (31)
LV increased wall thickness, *N* (%)	3 (30)	3 (30)	2 (15)	2 (15)
Depressed RVEF, *N* (%)	7 (70)	2 (20)	7 (54)	7 (54)
RV dilatation, *N* (%)	2 (20)	1 (10)	6 (46)	5 (38)

***A**: All patients with COVID-19 (N = 32) and Myocarditis (N = 22), **B**: only patients with follow-up with COVID-19 (N = 10) and Myocarditis (N = 13)*.

*T2W, T2 weighted; STIR, Short-TI Inversion Recovery; LGE, late gadolinium enhancement; WMA, wall motion abnormalities; LV, left ventricle; RV, right ventricle; EF, ejection fraction*.

All myocarditis cases (100%) fulfilled the updated LLC with evidence of myocardial oedema and non-ischaemic myocardial injury. Eight (25%) showed signs of pericarditis or pericardial effusion. Fifteen (68%) had systolic LV dysfunction and 13 (59%) LV dilatation. Impairment of RV function was present in 13 (59%) and RV dilatation in nine (41%) (Figures 4A,C, Table 3).

**Figure 5:**
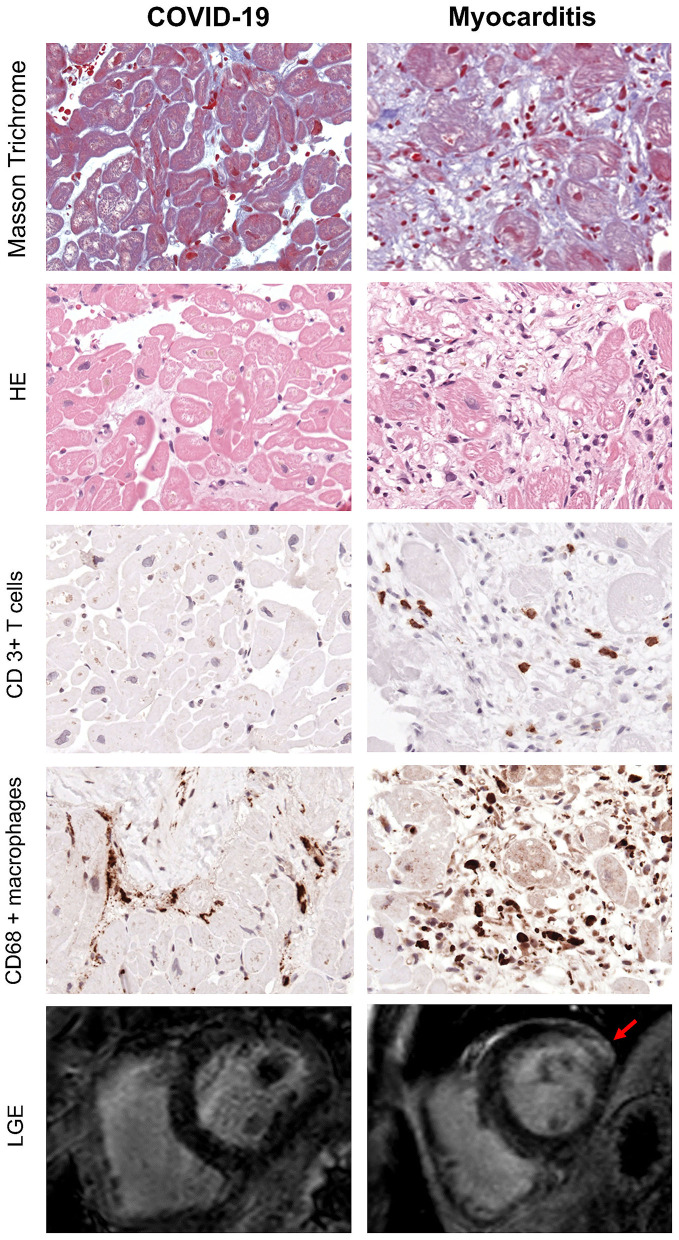
Exemplary EMB and CMR findings in COVID-19 (left) and Myocarditis (Right). First four rows from top represent respectively: Masson Trichrome, hematoxylin/eosin and immunohistological stainings of CD3+ T cells and CD68+ macrophages. In myocarditis, numerous T cells and macrophages are detected in presence of myocyte necrosis and fibrosis. In contrast, the majority of patients with COVID-19 often show fibrosis but no myocyte necrosis or significant T cell infiltration. However, the number of macrophages is enhanced (x400). Bottom row represents CMR short-axis late gadolinium enhancement (LGE) images: there is evidence of fibrosis in the lateral wall (indicated by the red arrow) in patients with myocarditis (right), while in COVID-19 there are no LGE positive areas in keeping with the absence of active inflammation and fibrosis indicated by the histology.

Endomyocardial biopsy (EMB) was available in 10 out of 32 post-COVID-19 patients. None of these samples presented evidence of acute or chronic myocarditis, as usually observed in viral myocarditis (Figure 4). In five (50%) of these patients, traces of previous myocardial inflammation were identifiable without evidence of ongoing inflammation *(* <14 infiltrating cells/mm^2^) (12). EMB was available in 15 out of 22 patients with classic myocarditis, 11 of 15 (74%) showed evidence of acute inflammation, and 2 of 15 (13%) had signs of chronic inflammation *(*Figure 5). In two patients with classic myocarditis on CMR, no histological evidence of inflammation was present (Figure 4B).

Of note, CMR LLC were positive in one patient (10%) with COVID-19 and in two patients (13%) with clinical suspicion of classic myocarditis, in whom EMB samples were negative (Figure 4B).

### CMR Parameters and WHO Classification

We compared the CMR parameters between patients with mild and moderate forms of the disease (as defined by WHO) and complicated or critical stages, respectively. Both LV and RV longitudinal systolic deformation is lower, and RV Mass and LV T1 native values are higher in patients with COVID-19 with complicated or critical disease compared with those with mild or moderate disease. LA and RA strains were lower in more severely affected patients. Additionally, there were trends for higher LVM and longer T2 times in complicated or critical compared with mild or moderate disease. Detailed results are presented in Table 4.

**Table 4:** WHO criteria of disease severity.

**COVID–19 Patients** **(***N*** = 32)**	**WHO Disease Severity Scale**		
	**WHO mild, moderate disease** **(***N*** = 20)**	**WHO severe disease** **(***N*** = 12)**	***P***
Initial Presentation			
Fever, *N* (%)	9 (45)	10 (83)	***0.033***
Chest pain, *N* (%)	6 (30)	2 (17)	0.40
Dyspnea, *N* (%)	12 (60)	8 (67)	0.71
Arrythmia, *N* (%)	0 (0)	1 (8)	0.19
Cough, *N* (%)	14 (70)	10 (83)	0.40
Nausea/Vomiting/Diarrhea, *N* (%)	8 (40)	3 (25)	0.39
Fatigue, weakness, *N* (%)	17 (85)	7 (58)	0.09
Amnesia, *N* (%)	6 (30)	4 (33)	0.99
Lack of taste or smell, *N* (%)	16 (80)	5 (42)	***0.027***
Persistent			
Fatigue/weakness, *N* (%)	9 (45)	0 (0)	***0.006***
Amnesia, *N* (%)	1 (5)	1 (8)	0.99
Lack of taste or smell, *N* (%)	2 (10)	0 (0)	0.26
Arrythmia, *N* (%)	6 (30)	4 (33)	0.84
**Center Ventricle**			
ED volume, mL/m2	78 ± 27	79 ± 23	0.85
ES volume, mL/m2	31 ± 22	34 ± 16	0.71
Stroke volume, mL/m2	50 ± 6	46 ± 10	0.19
Ejection fraction, %	64 ± 10	59 ± 11	0.23
Cardiac index, L/min/m2	3.5 ± 0.8	3.7 ± 1.2	0.50
Endo longitudinal strain %	–26.3 ± 4.1	–21.6 ± 4.3	***0.004***
Myo longitudinal strain %	–24.0 ± 3.3	−19.5 ± 3.8	***0.001***
Endo circumferential strain %	–32.8 ± 6.1	−31.0 ± 8.9	0.49
Myo circumferential strain %	–21.8 ± 3.8	−19.4 ± 4.5	0.13
LV Mass (g/m2)	50 ± 12	62 ± 25	0.07
**left Atrium**			
LA max Vol mL	37 ± 7	36 ± 11	0.84
LA emptying fraction %	65 ± 8	54 ± 15	***0.030***
LA strain %	42 ± 11	33 ± 12	***0.029***
**Right Ventricle**			
ED volume, mL/m2	77 ± 14	77 ± 17	0.97
ES volume, mL/m2	34 ± 8	39 ± 13	0.27
Stroke volume, mL/m2	43 ± 9	38 ± 7	0.16
Ejection fraction, %	55 ± 7	51 ± 9	0.14
Endo RV longitudinal strain %	–30.5 ± 5.9	–24.2 ± 9.1	***0.048***
Myo RV longitudinal strain %	–28.3 ± 5.3	–22.1 ± 8.9	***0.044***
RV Mass (g/m2)	12 ± 3	16 ± 5	***0.011***
**Right Atrium**			
RA max Vol mL	37 ± 12	37 ± 12	0.85
RA emptying fraction %	53 ± 11	55 ± 11	0.65
RA strain %	46 ± 11	36 ± 14	***0.034***
**Parametric Imaging**			
T1 native	1258 ± 55 (N=16)	1296 ± 30 (N=9)	***0.035***
ECV	26 ± 5 (N=16)	27 ± 3 (N=9)	0.64
T2	47 ± 4 (N=16)	51 ± 7 (N=9)	0.06
	(N=20)	(N=11)	P
**LGE No. (%)**			
Ischaemic	0 (0)	1 (9)	0.19
Nonischaemic	3 (15)	2 (18)	0.90
Pericardial	3 (15)	0 (0)	0.16
	(N=20)	(N=12)	
**Lake Louise Criteria**			
2018 LLC fulfilled, *N* (%)	0 (0)	3 (19) 5 (42) 4 (33)	
Myocardial edema	3 (15)		
Non–ischaemic myocardial injury	6 (30)		
Pericarditis/ pericardial effusion	3 (15)	5 (42)	
Systolic LV Dysfunctio*N* (global or regional)	2 (10)	6 (50)	
Depressed LVEF, *N* (%)	1 (5)	5 (42)	
LV dilatation, *N* (%)	2 (10)	1 (8)	
LV increased wall thickness, *N* (%)	2 (10)	5 (42)	
Depressed RVEF, *N* (%)	2 (5)	7 (58)	
RV dilatation, *N* (%)	2 (10)	2 (17)	

*WHO, World Health Organization; ED, end diastolic; ES, end systolic; Endo, subendocardial layer; Myo, mid–myocardial layer; LV, center ventricular; RV, right ventricular; LA, center atrial; RA, right atrial; ECV, extracellular volume; LLC, 2018 updated Lake Louise Criteria for acute myocarditis*.

*For comparison of the continuous variables, ANOVA and post–hoc Tukey's tests were used, for categorial variables Chi–square or Fischer tests test were used, a P < 0.05 was considered significant*.

*For incomplete set of data, N represents the number of subjects included in the analysis. Statistically significant p-values are indicated in bold*.

### Comparison of CMR Parameters Upon Follow-Up

**Figure 6:**
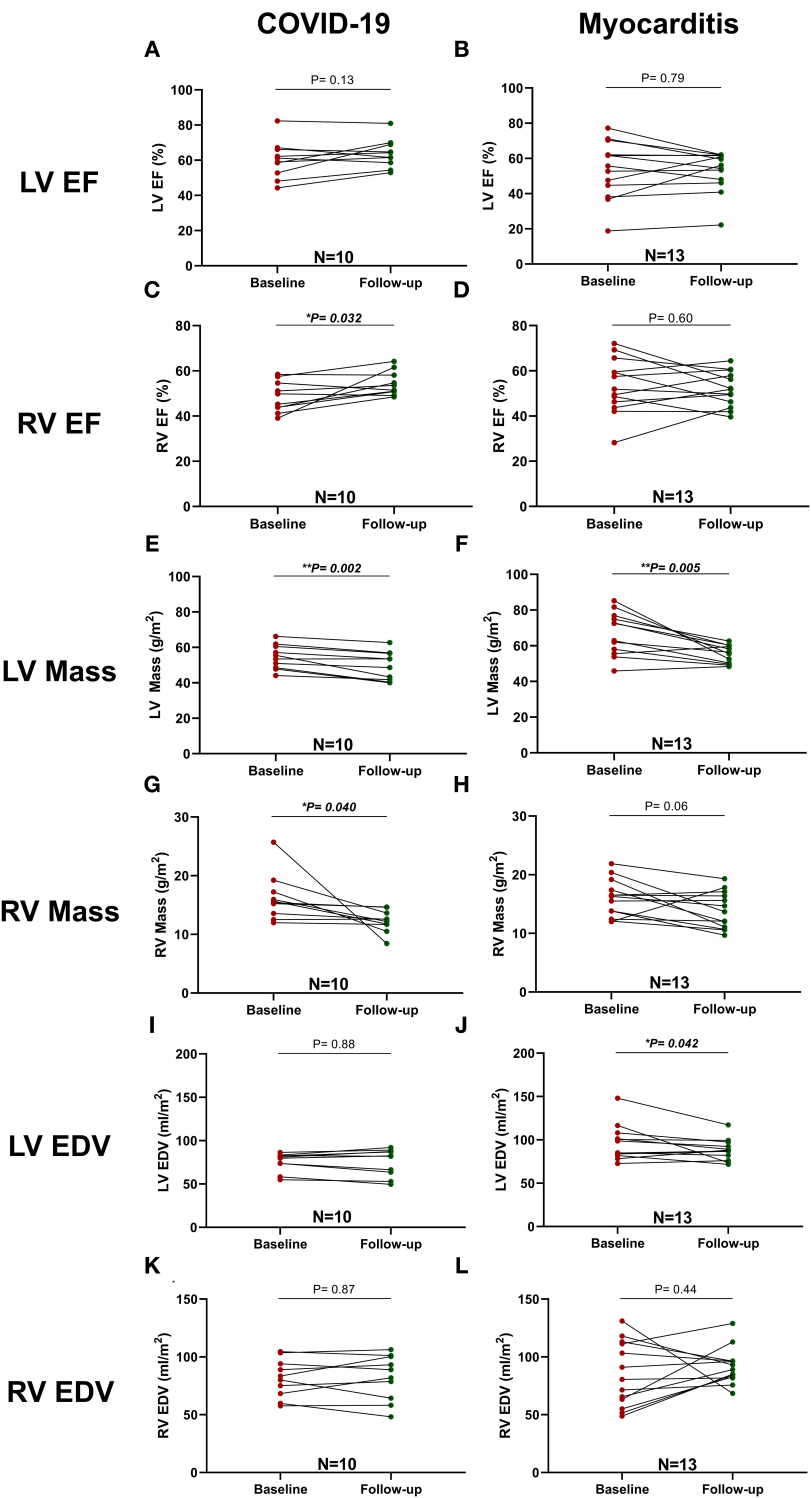
Comparison between LV and RV function and mass at baseline and follow-up in patients with COVID-19 and Myocarditis. On the left column, patients with COVID-19: **(A)** LV EF, **(C)** RV EF, **(E)** LV Mass, **(G)** RV Mass, **(I)** LV EDV, **(K)** RV EDV, and, on the right column, patients with Myocarditis: **(B)** LV EF, **(D)** RV EF, **(F)** LV Mass, **(H)** RV Mass, **(J)** LV EDV, **(L)** RV EDV. A *p* < 0.05 was considered statistically significant and indicated as follows: ^*^ < 0.05, ^**^ < 0.01, ^***^ < 0.001.

A complete description of these data is presented in Table 5. In post-COVID-19 patients, the difference in LVEF and Endo GCS (−31.7 ± 8.9 vs. −34.2 ± 8.6 %, *p* = 0.21) was not significant but showed a trend for improvement (60 ± 11 vs. 64 ± 8 %, *p* = 0.13). There was, however, significant improvement in Endo GLS (−23.4 ± 3.8 vs. −26.5 ± 3.7 %, *p* = 0.034) and in right ventricular ejection fraction (RVEF, 48 ± 7 vs. 54 ± 5 %, *p* = 0.032), whereas LV mass and RV mass decreased significantly (*p* = 0.002 and *p* = 0.040, respectively) (Figure 6).

**Table 5:** Cardiac magnetic resonance imaging findings follow–up.

	**COVID*****–*****19** ***N*** **=** **10**		***P***	**Myocarditis** ***N*** **=** **13**		***P***
	**Baseline**	**Follow–up**		**Baseline**	**Follow–up**	
**Left ventricle**						
ED volume, mL/m2	76 ± 11	75 ± 16	0.88	105 ± 35	94 ± 24	***0.042***
ES volume, mL/m2	31 ± 10	28 ± 10	0.10	52 ± 39	46 ± 25	0.24
Stroke volume, mL/m2	45 ± 8	47 ± 7	0.40	52 ± 13	48 ± 11	0.13
Ejection fraction, %	60 ± 11	64 ± 8	0.13	54 ± 16	53 ± 12	0.79
Cardiac index, L/min/m2	3.5 ± 1.0	3.2 ± 0.4	0.32	3.8 ± 1.0	3.1 ± 1.0	***<0.001***
Endo longitudinal strain %	−23.4 ± 3.8	–26.5 ± 3.7	***0.034***	–20.4 ± 7.4	–23.8 ± 4.3	***0.039***
Myo longitudinal strain %	–21.3 ± 3.0	–23.6 ± 3.2	0.07	–18.8 ± 6.5	–22.0 ± 3.9	***0.022***
Endo circumferential strain %	–31.7 ± 8.9	–34.2 ± 8.6	0.21	–25.4 ± 9.5	–25.8 ± 5.6	0.79
Myo circumferential strain %	–20.1 ± 4.0	–21.1 ± 4.1	0.35	–18.2 ± 7.1	–17.3 ± 3.7	0.56
LV mass (g/m2)	55 ± 7	50 ± 8	***0.002***	74 ± 27	59 ± 13	***0.005***
**Left atrium**						
LA max Vol mL	35 ± 9	33 ± 10	0.53	42 ± 13	38 ± 11	0.13
LA emptying fraction %	55 ± 15	65 ± 6	0.10	52 ± 20	60 ± 12	***0.026***
LA strain %	39 ± 14	39 ± 9	0.99	27 ± 18	32 ± 13	0.29
**Right ventricle**						
ED volume, mL/m2	82 ± 17	82 ± 20	0.87	85 ± 28	92 ± 16	0.44
ES volume, mL/m2	43 ± 12	38 ± 12	0.05	37 ± 14	44 ± 9	0.13
Stroke volume, mL/m2	39 ± 7	44 ± 8	***0.032***	44 ± 14	48 ± 11	0.22
Ejection fraction, %	48 ± 7	54 ± 5	***0.032***	53 ± 12	52 ± 8	0.60
Endo RV longitudinal strain %	–24.7 ± 8.0	–29.0 ± 5.9	0.32	–27.4 ± 8.6	–28.9 ± 5.5	0.41
Myo RV longitudinal strain %	–23.2 ± 8.1	–28.2 ± 6.1	0.26	–25.5 ± 8.1	–25.8 ± 5.3	0.84
RV Mass (g/m2)	16 ± 4	12 ± 3	***0.040***	16 ± 3	14 ± 3	0.06
**Right atrium**						
RA max Vol mL	37 ± 13	40 ± 17	0.27	45 ± 12	42 ± 11	0.34
RA emptying fraction %	55 ± 11	56 ± 9	0.94	48 ± 15	44 ± 9	0.46
RA strain %	39 ± 14	42 ± 13	0.70	36 ± 15	30 ± 10	0.14
**Parametric imaging**						
T1 native	1306 ± 39 (*n =* 8)	1257 ± 49 (*n =* 8)	***0.033***	1294 ± 24 (*n =* 5)	1224 ± 42 (*n =* 5)	***0.015***
ECV	26 ± 4 (*n =* 10)	25 ± 2 (*n =* 10)	0.27	28 ± 5 (*n =* 5)	26 ± 3 (*n =* 5)	0.31
T2	48 ± 6 (*n =* 8)	50 ± 5 (*n =* 8)	0.66	58 ± 10 (*n =* 10)	50 ± 5 (*n =* 10)	***0.043***
**LGE No. (%)**						
Ischaemic	1(10)	1 (10)	0.99	2 (15)	2(15)	0.99
Nonischaemic	2(20)	0 (0)	0.14	11 (85)	4 (31)	***0.015***
Pericardial	1 (10)	0 (0)	0.30	11 (92)	2 (15)	0.20

*ED, end diastolic; ES, end systolic; Endo, subendocardial layer; Myo, mid–myocardial layer; LV, left ventricular; RV, right ventricular; LA, left atrial; RA, right atrial; ECV, extracellular volume; LGE, late gadolinium enhancement*.

*For comparison of the continuous variables, paired Student t–test with Welch correction and, for categorial variables Chi–square test were used, a P < 0.05 was considered significant. Statistically significant p-values are indicated in bold*.

Similar differences in terms of GLS improvement and LV mass reduction were detected in the myocarditis subgroup (Figure 6).

**Figure 7:**
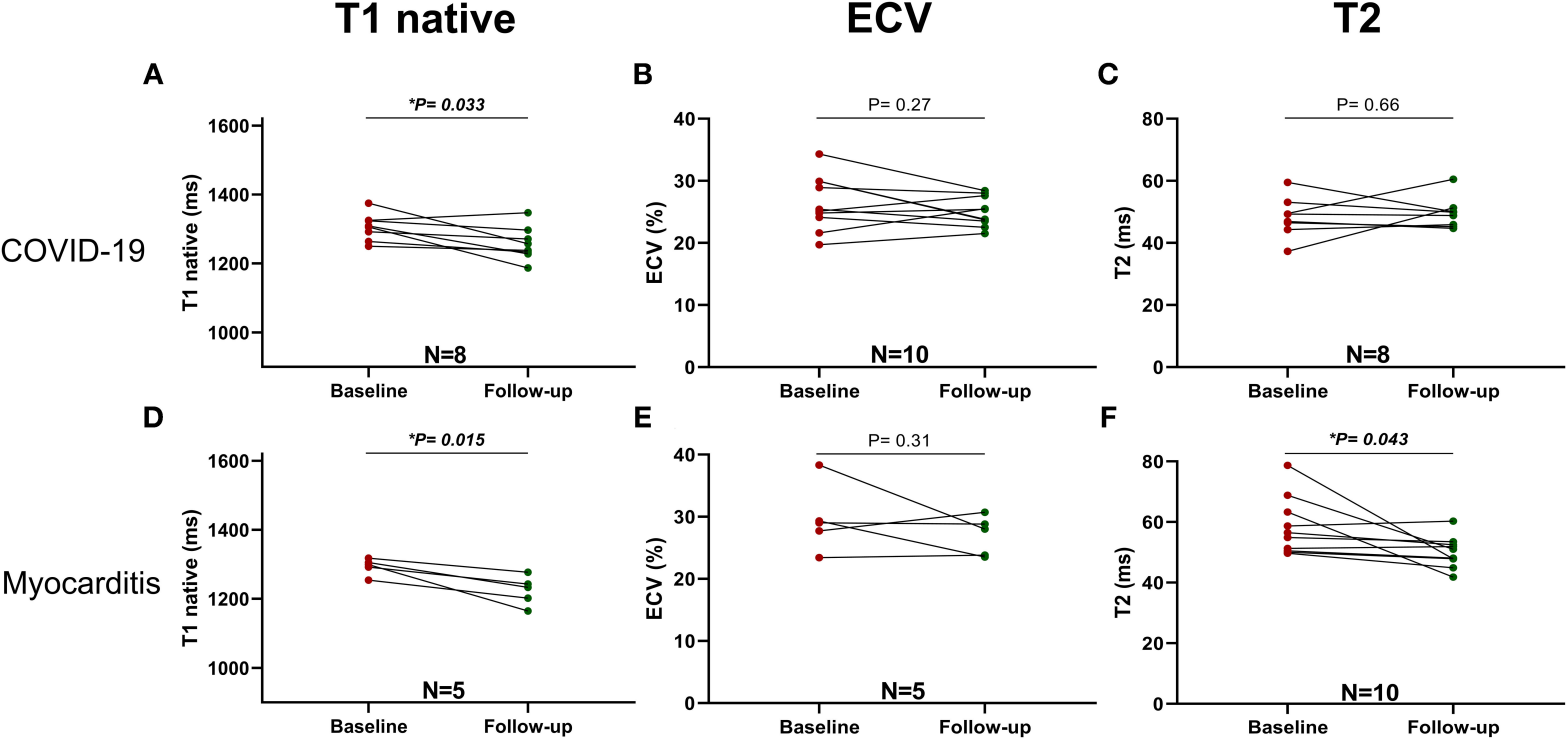
Parametric Imaging at baseline and follow-up in COVID-19 (upper row): **(A)** T1 native, **(B)** ECV and Myocarditis (lower row): **(C)** T2, **(D)** T1 native, **(E)** ECV, **(F)** T2. A *p* < 0.05 was considered statistically significant, and indicated as follows: ^*^ < 0.05, ^**^ < 0.01, ^***^ < 0.001.

**Figure 8:**
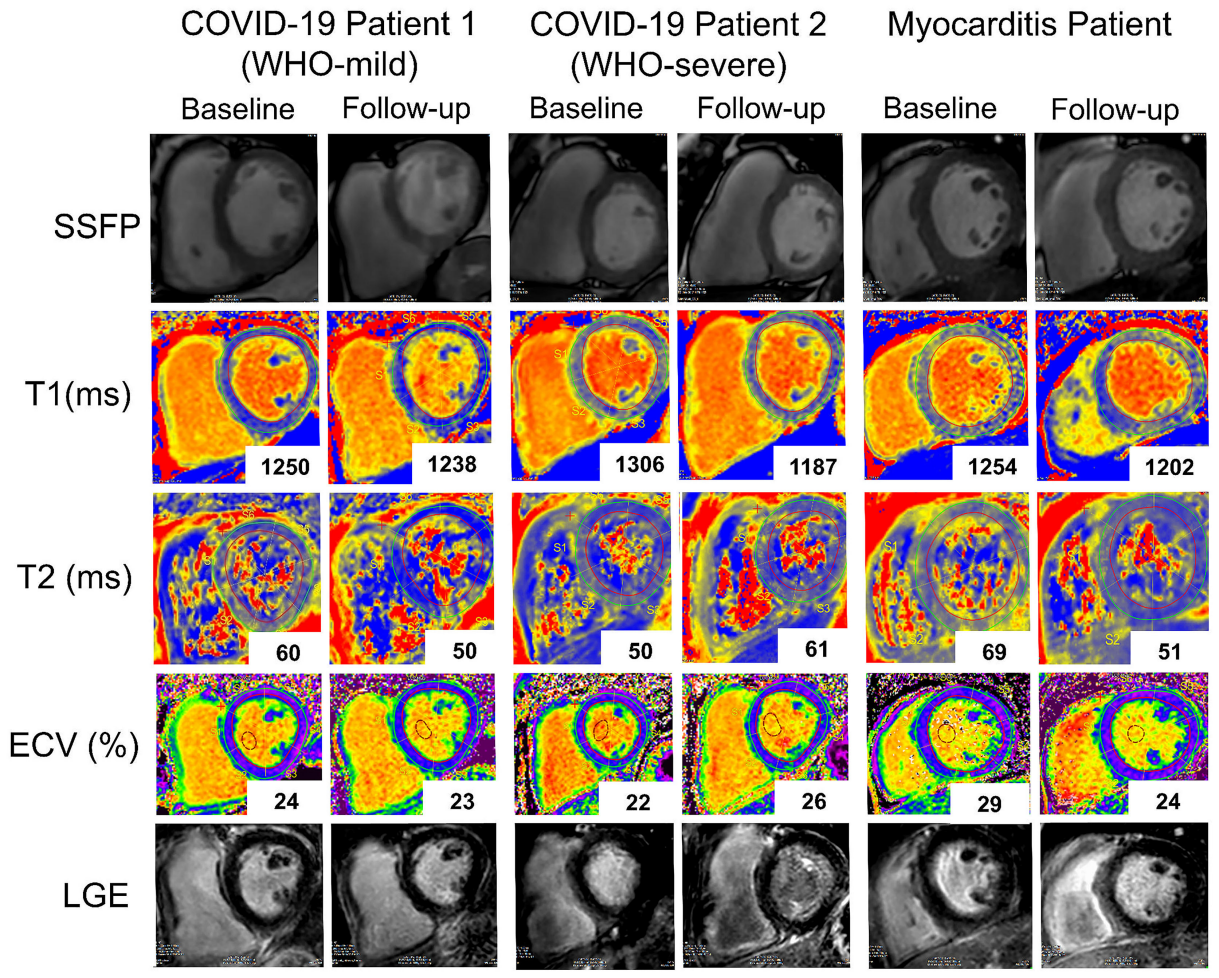
Representative baseline and follow-up CMR images of two patients with COVID-19 (COVID-19 patient 1 with a WHO-score: mild and COVID-19 patient 2 with a WHO-score: severe and a patient with Myocarditis. Rows (from top to bottom): SSFP-cine images, T1 native maps, T2 maps, Extracellular volume maps, late gadolinium enhancement images. While in the first patient with COVID-19 (first and second columns from the left), T1 native (from 1,250 to 1,238ms), and T2 (from 60 to 50 ms) signals improved at follow-up in the second patient with COVID-19 (third and fourth columns) T1 native signal improved (from 1,306 to 1,187 ms) while T2 (from 50 to 61ms) and ECV (from 22 to 26%) worsened. In the patient with myocarditis (fifth and sixth columns) there is at follow-up a marked improvement in all the parameters.

In addition, a significant reduction of native T1 values (1,306 ± 39 vs. 1,257 ± 94 ms, *p* = 0.033, *n* = 8) but not of ECV or T2 values was seen with COVID-19, whereas, in the myocarditis group, there was a significant reduction in T2 values (58 ± 10 vs. 50 ± 5 ms, *p* = 0.043, *n* = 10) and non-significant trends for reduction in native T1 values and ECV (Figure 7). Two examples of patients with COVID-19 and one with myocarditis to illustrate the complexity of structural modifications induced by the disease and the variability in recovery at follow-up are shown in Figure 8.

Dichotomized data (Figures 4D–F, Table 3) indicated that LLC criteria, fulfilled in two (20%) of the patients with COVID-19 at baseline, were fulfilled only in one (10%) at follow-up interval, with improvements in both myocardial oedema and myocardial injury from 4 (40%) to 2 (20%). Pericardial involvement was present in two (20%) of the COVID-19 patients, and, at follow-up, persisted only in 1(10%). Importantly, the RV function, impaired in seven (70%) of patients, remained impaired only in two (20%) of these patients at the follow-up visit. In patients with myocarditis, there was a marked improvement of myocardial inflammation at follow-up in majority of patients [from 13 (100%) to 6 (46%)] with five (38%) still fulfilling the LLC criteria at follow-up. The LV dysfunction [8 (62%)] and RV impairment [7 (54%)] persisted in all patients initially affected. Pericardial pathology improved in six patients [from 9 (69%) at baseline to 3 (23%) at follow-up] (Figures 4D–F, Table 3).

## Discussion

Our findings can be summarized as follows:

1. Patients with cardiac symptoms and a recent COVID-19 infection of varying severity showed only subtle changes in cardiac structure and function. On average, standard LV-ejection fraction and mass did not differ from controls, however, significant differences were observed with slightly elevated mean T2 relaxation times and decreased RV stroke volumes in COVID-19 patients.
2. In comparison, the morphological changes observed in COVID-19 patients were less pronounced than in patients with “classic” lymphocytic virus-associated myocarditis or eosinophilic myocarditis, the latter exhibiting marked myocardial and pericardial inflammation and injury in EMB and impaired RV- and LV-function on CMR.
3. In COVID-19 patients, a more severe clinical presentation (WHO severe or critical disease) was associated with lower biventricular longitudinal function, increased native T1 values and higher RV mass.
4. Only three (9%) of the COVID-19 patients fulfilled the diagnostic CMR criteria for acute myocarditis. More frequently, supportive criteria such as pericarditis and pericardial effusion (25%), LV (25%) and RV (28%) dysfunction were present and suggest a *sui generis* “myocarditis-like” pattern, the prognostic implications of which are yet to be established.
5. On EMB analyses, none of COVID-19 patients presented evidence of acute or persistent inflammation, in contrast, the majority of myocarditis patients [13 of 15 (87%)] showed ongoing myocardial inflammation.
6. In COVID-19 patients, LV GLS, LVM, RV EF, T1 values mildly improved at follow-up while T2 values remained elevated.

Although we found that cardiac function on the whole was unaffected in patients who recovered from a SARS-CoV-2 infection, we found significant functional impairment in a small subset of patients. Out of 32 patients, eight (25%) showed global or regional LV dysfunction, nine (28%) showed depressed RV function, 10 (31%) showed structural myocardial alterations, and eight (25%) showed pericardial effusion or pericarditis on CMR. Although these changes seem to recede over time and some of might have partially resolved at the time of the first scan after the acute phase of a COVID-19 infection, pathologic cardiac findings could be more severe in patients with pre-existing cardiac disease, in particular, heart failure (26). Particularly, RV remodeling, diagnosed and assessed with transthoracic echocardiography, increased the mortality risk in patients with COVID-19 by more than 100% (27). Using CMR, we showed that decreased RV function is present in about one in four post-COVID-19 patients [9 (28%)]. In half of whom [4 (13%)], RV dilatation is also present.

In agreement with recent data (9), we found that elevated myocardial T2 relaxation times in patients with COVID-19 did not recede at follow-up. This is possibly related to a certain degree of reactive myocardial inflammation triggered by an abnormal immune response that persists even months after an infection (28). However, since almost all other functional markers improve over time, the clinical significance of this finding remains unclear and merits further investigation in future long-term studies.

Previous reports signal the particular targeting of the endothelium by the SARS-CoV2 (4, 29), especially in cases with systemic severe disease. Reduced longitudinal function of the heart is a primary hallmark of a dysfunctional myocardial microvasculature (30). Our findings indicate that both LV and RV long-axis deformation are decreased in more severe forms of COVID-19. This corresponds to increased values of native T1 relaxation times in these patients, which is a marker of persistent inflammatory response that is possibly accompanied with diffuse structural changes. Taken together, these findings may provide a link to capillary endothelial damage in patients with more severe forms of COVID-19. All patients with a recent COVID-19 infection included in this study improved clinically over time and attended the CMR examinations in an ambulatory outpatient setting. Thus, more pronounced myocardial injury may be present in a more acute stages of the disease in older patients and in patients with underlying cardiac conditions. Impaired RV function that can be observed, in particular, in patients with COVID-19 with a more severe initial presentation or clinical evolution (WHO Score of 3 or 4) may be due to a persistent lung inflammation with microvascular congestion and retrogradely elevated pulmonary arterial pressure (PAP), which could not be excluded by our study where contemporaneous high resolution CT chest imaging was not available.

In a multi-center analysis of 68 hospitalized patients that succumbed to COVID-19 in Wuhan, China, extensive myocardial damage was identified as the main cause of death in 5 (7%) of the cases (31). In contrast, the largest whole heart study to date (32) examined explanted hearts from 39 patients who died following a COVID-19 infection identified the intra-myocardial presence of the virus in 24 samples and signs of viral replication in five samples but failed to demonstrate the presence of any acute inflammatory infiltration of the myocardium even in patients with clinically significant viral load (>1,000 copies). In a similar study, an unexpectedly high density of macrophages was identified in the cardiac tissue of a majority of patients who died of COVID-19, and overt lymphocytic myocarditis was identified in 14% (14) We also observed increased amounts of CD68+ macrophages but not of CD3+ lymphocytes or other specific immune cells in our post-COVID-19 patients. However, it remains uncertain if these dire consequences are the direct effects of viral penetration of the cardiac structures and intra-myocardial viral replication or rather part of an exacerbated systemic response, such as autoimmune virus-triggered cytokine storm or sepsis (33).

In our study, none of the EMB samples obtained from patients with COVID-19 showed any sign of acute or chronic lymphocytic inflammation or viral RNA in the myocardium. In contrast, 13 (87%) out of 15 patients with myocarditis showed histologic evidence of acute inflammation or clinically relevant virus presence in the myocardial tissue in EMB. However, importantly, one (10%) of the post-COVID-19 patients and two (13%) of the patients with myocarditis showed positive CMR LLC criteria but had a negative EMB sample (Figures 4A,B). Our results are in agreement with a recently published meta-analysis (34), scrutinizing 277 post-mortem histopathology reports in COVID-19 cases, which identified a very low prevalence of myocarditis if strict diagnostic criteria were applied. However, some myocardial abnormalities were present in as many as half of the cases.

Our study provides further evidence for the role of CMR in the diagnosis of cardiac complications in patients with COVID-19. Yet, due to the small number of included patients, the results of our study should be interpreted with caution (11). Nonetheless, we believe that our study shows that a greater number of post-COVID patients would benefit from a comprehensive CMR (13) work-up and should ideally be included in multi-center, national, or international CMR COVID-19 databases with stringent long-term follow-up. Patients included in these longitudinal studies should include patients with pre-existing cardiovascular disease and risk factors, in whom myocardial injury and dysfunction may prove to be more severe, recovery slower, and long-term sequelae more pronounced. In addition, our study also underlines the complex and incomplete overlap of CMR and EMB criteria in the diagnosis of acute myocarditis and suggests their complementary diagnostic role (33).

Several studies (35–39) agree on the fact that acute myocarditis or myocarditis-like traits are present after anti-SARS-CoV2 vaccination in a minority of subjects with CMR findings similar to those observed in patients with COVID-19. However, the mechanisms of these change as the mechanisms of post-COVID-19 myocardial modification remain largely unknown. In addition, while it is legitimate to suppose that pathophysiological similarities related to the specific immune response elicited SARS-CoV2 viral particles, more focused studies are warranted to demonstrate such correlations.

Immunosuppressive therapy was shown to be effective in the partial recovery of cardiac function in patients with chronic myocarditis or HF resulting from resolved acute myocarditis. (40) RECOVERY Trial demonstrated the beneficial effect of Dexamethasone in severely affected patients hospitalized for COVID-19 (41) and several other immunomodulatory interventions (42–44) mostly done in an acute setting and in very ill patients which improved mortality rate and clinical course of the disease. HEAL-COVID clinical trial (https://clinicaltrials.gov/ct2/show/NCT04801940), which commenced in April 2021, aims to recruit subjects recovered from COVID-19 who experience longer-term complications of the disease. So far, the lack of consensus regarding the molecular pathophysiology of these changes and their reversibility hampers a more targeted pharmacological approach. With the paradigm of long-COVID now widely accepted, we expect to see an increase in the number of clinical trials including incompletely recovered patients, including those with persistent myocardial or pericardial disease.

## Limitations

COVID-19 is an ongoing global pandemic, and study findings to support clinical guidelines and decision making are urgently needed. Our study was primarily designed to assess what appears to be the most important alteration observed with CMR in post-COVID-19 patients, namely, parametric mapping (T1, T2 relaxation times). T1 and T2 values notoriously vary between scanners from different manufacturers and field strengths. Thus, in order to increase the sensitivity, the comparison of parametric mapping between the three groups included only patients and volunteers scanned with the same 3T Philips Ingenia scanner. To comply with these inclusion criteria, the final number of patients included in the study and the number of these patients who underwent a follow-up scan were relatively reduced. Importantly, we acknowledge the age disparity between healthy controls, post-COVID-19, and myocarditis groups. There was more variability in the follow-up interval within in the myocarditis group compared to the post-COVID-19 group due to a more complex clinical management, frequently involving hospitalization, and clinically indicated multiple scans. Despite the best of our efforts, some of the datasets remain incomplete, in particular, clinical data collected retrospectively from patients with COVID-19 and patients with myocarditis. To overcome this limitation, we clearly indicated the exact number of datasets available per subgroup. As our study design did not permit the examination of hyper-acute cardiac manifestations of COVID-19, our main purpose was to investigate whether persistent cardiac changes, as proposed previously, can induce structural or functional remodeling of the heart and impede complete recovery.

## Conclusions

In our cohort, CMR and EMB findings revealed that a SARS-CoV-2 infection shows relatively mild but variable cardiac involvement. More symptomatic patients and those with higher clinical care demands are more likely to exhibit impaired myocardial function and chronic inflammation compared to patients with “classic” acute myocarditis during the acute and convalescent phases. Our study highlights the importance of collecting large multicentre cardiac imaging data from patients with and recovering from COVID-19.

## Data Availability Statement

The original contributions presented in the study are included in the article/supplementary material, further inquiries can be directed to the corresponding author.

## Ethics Statement

The studies involving human participants were reviewed and approved by the Charité – Universitätsmedizin Berlin Ethics Committee and comply with the Declaration of Helsinki.

## Author Contributions

RT and SK designed the study. RT, PD, CG, VZ, JW, and JE participated in data collection and analysis. PD, AF, AB, SK, DG, and TC recruited the patients and supervised the CMR scans. KK and CT supervised the histology analyses and reviewed the EMB results. GK, TK, TC, PS, MW, KK, SV, BP, CT, and SK reviewed the manuscript. All authors contributed to the article and approved the submitted version.

## Conflict of Interest

CS was employed by the company Philips Healthcare Systems. The remaining authors declare that the research was conducted in the absence of any commercial or financial relationships that could be construed as a potential conflict of interest.

## Publisher's Note

All claims expressed in this article are solely those of the authors and do not necessarily represent those of their affiliated organizations, or those of the publisher, the editors and the reviewers. Any product that may be evaluated in this article, or claim that may be made by its manufacturer, is not guaranteed or endorsed by the publisher.

## Abbreviations

AHA: American Heart Association
AUC: area under curve
bSSFP: balanced steady state free precession
CMR: cardiac magnetic resonance
COVID-19: Coronavirus disease 2019
DNA: desoxyribonucleic acid
ECV: extracellular volume
EF: ejection fraction
EMB: endomyocardial biopsy
ESC: European Society of Cardiology
FT: feature tracking
GCS: global circumferential strain
GLS: global longitudinal strain
HF: heart failure
LA: left atrium/atrial
LGE: late gadolinium enhancement
LLC: Lake Louise Criteria
LV: left ventricle/ventricular
LVM: left ventricular mass
NT-proBNP: N-terminal pro-B type natriuretic peptide
RNA: ribonucleic acid
RV: right ventricle/ventricular
SARS-CoV-2: severe acute respiratory syndrome coronavirus 2
TE: echo time
TR: repetition time.
